# Scalable radiotherapy data curation infrastructure for deep-learning based autosegmentation of organs-at-risk: A case study in head and neck cancer

**DOI:** 10.3389/fonc.2022.936134

**Published:** 2022-08-29

**Authors:** E. Tryggestad, A. Anand, C. Beltran, J. Brooks, J. Cimmiyotti, N. Grimaldi, T. Hodge, A. Hunzeker, J. J. Lucido, N. N. Laack, R. Momoh, D. J. Moseley, S. H. Patel, A. Ridgway, S. Seetamsetty, S. Shiraishi, L. Undahl, R. L. Foote

**Affiliations:** ^1^ Department of Radiation Oncology, Mayo Clinic Rochester, Rochester, MN, United States; ^2^ Department of Radiation Oncology, Mayo Clinic Arizona, Phoenix, AZ, United States; ^3^ Department of Radiation Oncology, Mayo Clinic Florida, Jacksonville, FL, United States

**Keywords:** curation, artificial intelligence, deep learning, convolutional neural network, autosegmentation, radiotherapy, head and neck cancer, organs-at-risk

## Abstract

In this era of patient-centered, outcomes-driven and adaptive radiotherapy, deep learning is now being successfully applied to tackle imaging-related workflow bottlenecks such as autosegmentation and dose planning. These applications typically require supervised learning approaches enabled by relatively large, curated radiotherapy datasets which are highly reflective of the contemporary standard of care. However, little has been previously published describing technical infrastructure, recommendations, methods or standards for radiotherapy dataset curation in a holistic fashion. Our radiation oncology department has recently embarked on a large-scale project in partnership with an external partner to develop deep-learning-based tools to assist with our radiotherapy workflow, beginning with autosegmentation of organs-at-risk. This project will require thousands of carefully curated radiotherapy datasets comprising all body sites we routinely treat with radiotherapy. Given such a large project scope, we have approached the need for dataset curation rigorously, with an aim towards building infrastructure that is compatible with efficiency, automation and scalability. Focusing on our first use-case pertaining to head and neck cancer, we describe our developed infrastructure and novel methods applied to radiotherapy dataset curation, inclusive of personnel and workflow organization, dataset selection, expert organ-at-risk segmentation, quality assurance, patient de-identification, data archival and transfer. Over the course of approximately 13 months, our expert multidisciplinary team generated 490 curated head and neck radiotherapy datasets. This task required approximately 6000 human-expert hours in total (not including planning and infrastructure development time). This infrastructure continues to evolve and will support ongoing and future project efforts.

## Introduction

Radiotherapy (RT) clinical practice is deeply and culturally rooted in technology and technological innovation. The RT community has benefited from many major advances in terms of 1) RT modalities (fundamental types of electromagnetic radiation or subatomic particles, or radiation delivery technique used); 2) the 3D imaging used as the basis for RT planning and delivery; and 3) the supporting computational infrastructure requirements for treatment planning, delivery and monitoring. The modern era of Big Data and outcomes-driven medicine promises an increasingly nimble, patient-centered RT approach. However, this requires increasingly sophisticated and integrated tools for cancer diagnosis and staging, RT treatment planning, delivery, monitoring and plan adaptation. At the same time, many inefficiencies persist in current RT workflows related to disjoining technologies, our human-expert (physician, physicist, dosimetrist, therapist) interventions and interdependencies of human-expert task completion. These inefficiencies create bottlenecks wherein our human lack of scalability is a (if not, “the”) major limiting factor – which is counterproductive to nimble decision-making and adaptability.

Artificial Intelligence (AI) promises to address many persistent RT workflow bottlenecks. In the last 25 years, the RT community has witnessed a renaissance in the evaluation and adoption of machine learning (ML)-based solutions in many aspects of RT patient care, from prognostic methods in the patient outcomes research realm including radiomics, to clinical adoption of platforms for automated or semi-automated RT plan generation and related quality assurance (QA) (1–10). More recently, we have benefited from a virtual explosion in the application of deep convolutional neural networks (hereafter referred to as deep learning, or DL) for tackling complex imaging-related problems. In the context of RT, arguably the best-realized example of DL has been organ-at-risk (OAR) CT and MRI segmentation used as a starting point for the RT planning process (1, 4, 6, 11–21). Currently, numerous vendors provide commercial autosegmentation solutions. The number of vendors is expanding rapidly which indicates the computational practicality of autosegmentation algorithms (14) as well as the increasing demand for safe, effective and efficient autosegmentation solutions.

DL model training for OAR segmentation requires input voxel-labeled image data, with unique labels corresponding to each organ which is modeled independently. With current DL training methods, the performance or accuracy of DL tools for OAR segmentation depends critically on the quality of these inputs, i.e., dataset “curation,” focusing particularly on 1) how representative the training images are of current and projected future practice; 2) quantity of training datasets and 3) consistency of training labels. Numerous recent publications have reiterated critical needs in terms of dataset curation and/or general awareness of the AI state-of-the-art and its limitations (4–6, 11, 13, 19, 22, 23). Somewhat paradoxically, published literature is extremely sparse in terms of describing infrastructure requirements, methods and related best practices for data curation.

Working at an enterprise (or health system-wide) level in our respective radiation oncology departments within Mayo Clinic, we have recently engaged collaboratively with an external partner to develop custom DL-based OAR autosegmentation. Thus, working as a multidisciplinary dataset curation team and connected across multiple campuses in the U.S., we are actively engaged in RT dataset curation on a large scale. The present report outlines our curation efforts and infrastructure developed to date, focusing mainly on our first specific use-case of preparation of expert OAR segmentations (“contours”) for head and neck (H&N) malignancies.

## Materials and methods

### Personnel resources

The production of a high-quality DL model for OAR segmentation requires careful planning, coordination of various personnel roles and maintenance of consistent quality. All this tends to require significant human-expert effort. The H&N dataset curation team for this project was comprised of stakeholders representing five disciplines, namely an information technology specialist with software development expertise [author SSe], two expert radiation oncologists (RO) who focus on management of H&N cancer [authors RLF, SHP], five therapeutic medical physicists [authors AA, DJM, SSh, ET], two certified medical dosimetrists with expertise in H&N treatment planning and normal human anatomy on CT and MRI [authors AH, AR] and five Medical Dosimetry Assistants (MDAs), who we define in the Rochester (Minnesota) practice (only) as certified radiation therapists receiving specialized, in-house training in RT-image data handling, image fusion and OAR segmentation on CT and MRI. In preparation for this project, the MDAs involved in this work [authors JC, NG, TH, RM, LU] received focused training in H&N OAR CT segmentation from an expert dosimetrist [author AH] and expert RO [author RLF].

### Curation dataset, format and objects

Summarized in **Table 1** are the data/objects included in the “curated dataset” and which were defined by a broader set of project goals established between Mayo Clinic and our external partner. In general, DL model training for OAR segmentation requires 3D imaging along with OAR labels defined on the same spatial grid (i.e., on the same imaging voxels). The training image sets for autosegmentation were H&N planning CTs in DICOM format (24), chosen from patients who previously received H&N RT at Mayo Clinic. The majority of these planning CTs were produced from iterative reconstruction methods for metal artifact reduction, which improves image quality in the context of dental fillings/hardware or other metallic surgical implants (which are not uncommon in postoperative H&N cancer patients). H&N OAR segmentations on these images were reviewed, revised or re-created as necessary to a consistent standard by the expert personnel as DICOM “structure sets” [i.e., in DICOM-RT-Struct format (25)] which are vectorized, or represented as sets of contiguous 2D contours drawn on the defined CT slices (per OAR). Additional DICOM CT reconstructions, acquired as part of the same/original CT planning study were also included, namely a “Small Field-of-View” (SFOV) reconstruction (which had higher spatial resolution over an anatomically truncated field-of-view and was derived from the same raw data as the original planning CT) and an additional CT acquired post IV contrast, if available. In some cases, a contrast-SFOV reconstruction was also included. These alternate CT scans are reflective of current institutional standard-of-care and are important for accurate delineation of small anatomic H&N structures, which tend to present with limited CT contrast. Including the alternate CT series in the curated dataset was necessary for evaluation and recontouring by the expert MDAs and ROs, regardless of whether they were used in DL model training. The curated dataset also included previously treated radiation plans (DICOM-RT Plan) with associated 3D dose matrices (DICOM-RT Dose) and structure set objects. This data could facilitate the exploration of DL applied to RT planning (although this broader topic is out of scope for the present work).

**Table 1:** DICOM objects comprising the curated datasets.

Description	DICOM modality	Required (R) vs. Optnl. (O)	Typical voxel size (x,y,z) in mm
H&N Curated OARs	RTSTRUCT	R	*n/a*
H&N Planning CT Recon.	CT	R	(1.27, 1.27, ≤2.5) *≠*
Small FOV CT Recon.	CT	O	(≤0.59, ≤0.59, ≤2.5) *†*
Contrast CT Recon.	CT	O	(1.27, 1.27, ≤2.5) *≠*
Contrast CT Registration	REG	O	*n/a*
H&N Clinical OARs	RTSTRUCT	R	*n/a*
H&N Clinical RT Plan	RTPLAN or RTIONPLAN	R	*n/a*
H&N Clinical RT Dose	RTDOSE	R	(≤3.0, ≤3.0, ≤3.0)

≠ Most-frequent voxel size in z was 2 mm.

† Most-frequent voxel size was (0.59, 0.59, 1) mm. n/a, not applicable.

### Retrospective (representative) dataset identification

As summarized in **Table 2**, the expert H&N ROs provided a broad set of relevant disease sites (or sub-sites) for possible inclusion in our curated dataset. Based on this information, we identified patients for potential inclusion using an automated web-based query of our department database of patient demographics, diagnosis, pathology, staging, treatment planning information and outcomes (“Outcomes Database”) (26). We did not exclude cases based on race, sex, age or ethnicity. The Outcomes Database query enabled us to extract the patients’ institutional medical record numbers (MRNs) and relevant RT treatment course information, including explicit identification of the treated DICOM-RT Plan Unique Identifier (DICOM UID). This allowed for identification and downstream collection of associated DICOM objects prior to the automated DICOM de-identification (DeID) process (to be described).

**Table 2:** H&N cancer sites or histologies for cases included in our curated dataset.

H&N Cancer site	Sub-sites or Relevant histologies (if applicable)
Hypopharynx	Pyriform sinus; postcricoid mucosa; posterior pharyngeal wall
Larynx	Supraglottic; glottic; subglottic
Nasal cavity	
Nasopharynx	
Oral cavity	Lip; gingiva; buccal mucosa; floor of mouth; oral tongue; retromolar trigone; hard palate
Oropharynx	Tonsil; base of tongue; soft palate; posterior pharyngeal wall
Paraganglioma	Carotid body; vagale; jugulotympanicum
Paranasal sinus	Maxillary; sphenoid; ethmoid; frontal
Salivary gland	Parotid; submandibular; sublingual; minor
Skin	Squamous cell ca.; basal cell ca.; Merkel cell ca.; melanoma
Thyroid gland	

In addition to the DICOM files included in the curated dataset, de-identified (DeID) H&N patient demographic data was separately prepared to allow for DL model bias minimization applied to our external collaborator’s process of identification of model training subgroups (specifically, the data partitions for training, validation and independent testing). This information necessarily included patient age at RT, race, sex, U.S. zip code of residence (for possible correlation with prevalence of economic disparities) (27), diagnoses and surgical status at RT.

Given our downstream use and sharing of only DeID data, this project was deemed as qualifying for a human subjects’ research exemption by the Mayo Clinic Institutional Research Board. The state of Minnesota requires Mayo Clinic patients seen in Rochester, Minnesota (or its affiliates), to give explicit permission to use their data for any research purposes. Note that patients from the European Union were excluded *a priori* so as not to conflict with General Data Protection Regulation (28).

### OAR standardization

**Table 3** summarizes the H&N OARs being included in the curated DICOM-RT Struct (per patient), reflecting a comprehensive total of 42 individual organs. As mentioned in the Introduction, standardization in terms of OAR definition is ultimately crucial for accuracy in DL-based modeling. The benefits of OAR standardization extend well beyond DL model training and touch on many aspects of RT quality, inclusive of practice uniformity and multi-institutional clinical trials research (inter-institutional RT plan and outcomes comparability). Fortunately, Mayo Clinic has recently standardized its definitions for OAR segmentation across its radiation oncology clinics. These Mayo Clinic enterprise H&N contouring guidelines largely follow contemporaneous consensus guidelines (29). As is not uncommon elsewhere in the RT community, the Mayo Clinic guidelines include institution-specific modifications for certain regional-anatomical organ groupings or organ sub-portions such as the oral cavity, nasal cavity and the cervical esophagus, and include OARs not included in consensus guidelines such as the mastoid air cells and external auditory canals

**Table 3:** H&N OAR segmentation labels and their anatomic designation.

H&N OAR labels	Anatomic name or designation
brachial_plex_[l,r]	Brachial plexus nerve
brain	
brain_stem	Brainstem
carotid_artery_[l,r]	
cochlea_[l,r]	
constrictors_p	Pharyngeal constrictor muscle
cord	Spinal cord
crico_p_inlet	Cricopharyngeal inlet muscle
esophagus	
esophagus_cerv	Cervical esophagus
ext_aud_canal_[l,r]	External auditory canal
eye_[l,r]	
lacrimal_[l,r]	Lacrimal gland
larynx	
lens_[l,r]	
lips	
lung_[l,r]	
mandible	
mastoid_[l,r]	Mastoid air cells
nasal_cavity	Nasal cavity (region)
optic_nrv_[l,r]	Optic nerve
oral_cavity	Oral cavity (region)
parotid_[l,r]	Parotid gland
pituitary	Pituitary gland
retina_[l,r]	
semi_cir_canal_[l,r]	Semicircular canal
sub_mandib_[l,r]	Submandibular gland
thyroid	Thyroid gland

### Technical infrastructure for curation efforts

**Figure 1:**
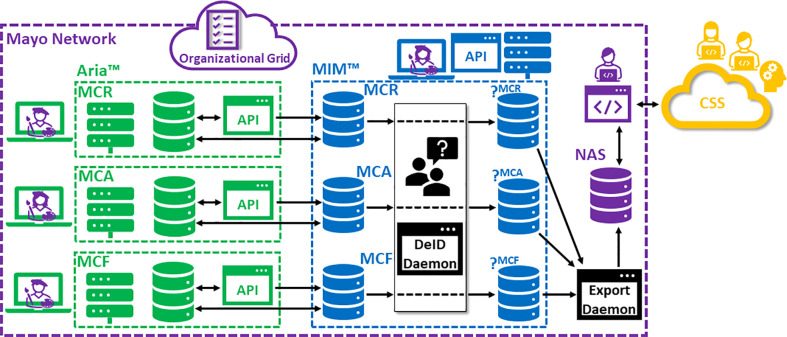
Schematic of technical curation infrastructure. (MCR, Mayo Clinic Rochester; MCA, Mayo Clinic Arizona; MCF, Mayo Clinic Florida; DeID, de-identification; CSS, Cloud Storage Service).

**Figure 1** is a schematic of our technical curation environment for generating, storing, accessing and manipulating the DICOM objects comprising the curated dataset. Also included in this figure are indications of how the DICOM data flows through the system. Partitioning exists between native and DeID DICOM storage. Also note that DICOM studies being derived from individual Mayo Clinic campuses (Rochester, MN [MCR], Phoenix, AZ [MCA] and Jacksonville, FL [MCF]) are siloed until the point of archival on Network-Attached Storage (NAS). In general, this design, leveraging both a distributed RT Information system (Aria™, Varian Medical Systems, Inc., Palo Alto, CA) as well as a distributed DICOM-RT-aware distributed Picture Archiving and Communications System (PACS) (MIM Software Inc., Columbus, OH), provides 1) accessibility (with opportunities for granular access control); 2) scalability; 3) intrinsic and transparent data organization; 4) data redundancy (in case of loss) and 5) opportunities for automation (e.g., custom user scripts) and custom interfacing (e.g., *via* DICOM adaptors).

#### Cloud-based data and workflow organizational grid

Important for coordination of curation efforts and stakeholder communication was the use of an organizational grid (i.e., a spreadsheet). Modern cloud-based document management tools allow for both file access control and multi-user file access, with real-time file updates being applied dynamically to all users’ instances. This powerful functionality was leveraged for this project. Within its private network, the Mayo Clinic distributes Microsoft (MS) Office365™ web applications (via SharePoint™) built on top of OneDrive™ cloud storage (Microsoft, Inc., Redmond, WA). In our case, a single multiple-tabbed MS Excel™ spreadsheet was used to coordinate progress per curated case. Excel tabs were used to separate data coming from each campus; individual cases were organized in rows with columns used to indicate per-case progress through the below-described steps.

#### Distributed Aria™/Citrix™ environment and automatic data extraction

As mentioned above, Mayo Clinic’s RT information system is Aria™ (Version 15.1, Varian Medical Systems, Inc.), deployed across the Mayo campuses *via* thin clients using Citrix™ technology (Citrix Systems, Inc., Fort Lauderdale, FL). For network performance and data security reasons, each of our three campuses has its own Aria-over-Citrix™ Application Server farm, where the MCR infrastructure also handles the broader Midwest Regional (Minnesota-Wisconsin) radiation oncology practice.

Aria supports both an application programming interface (API) to its database (for standalone applications and interfaces) as well as an API to its treatment planning system (Eclipse™), or ESAPI, which is user-runtime context driven. Our Mayo Clinic Radiation Oncology IT Operations team has developed expertise in building and developing in-house tools leveraging these Aria APIs. For this project, a tool dubbed the “Extractavator” was created in-house to automatically extract the set of objects comprising the curated dataset from the Aria database for the existing patient and DICOM Frame-of-Reference (coordinate system) defined by the structure set loaded into context in the Eclipse workspace. The Extractavator automatically sends this contextually referenced data to the given MIM-PACS patient list established for each Aria data source. This extraction operation is executed in the background, allowing curators freedom to resume work on other tasks within the same instance of Eclipse.

#### Distributed MIM™/Citrix™ environment

MIM Maestro™ (MIM Software, Inc., Cleveland, OH) deployed over Citrix™ extended access to DICOM data stored on a central MIM–PACS server. This distributed MIM environment served as the main hub for our data curation processes once the DICOM data was extracted from Aria. MIM has several relevant capabilities, namely: 1) its user-friendly and feature-rich DICOM image display and manipulation toolset, particularly as it relates to image registration; 2) its DICOM-RT specific toolset, allowing users to display and manipulate RT objects such as DICOM-RT Dose, (DICOM-RT Plans) and DICOM-RT Structs; 3) its in-built support of customized automation, where this capability can be initiated by users within the users’ runtime environment using a flexible combination of MIM Workflows™ (i.e., user scripts using MIM-supported operations or methods) and MIM Extensions™ (i.e., user code leveraging an API), or by the MIM-PACS server *via* the MIM Assistant™ service. The latter allows for MIM administrators to define DICOM-data-driven “Assistant Rules” that can be triggered in numerous fashions from user-defined criteria. Assistant Rules can also incorporate MIM Extensions™ directly or can execute MIM Workflows by instantiating a non-interactive instance of MIM Maestro™ in the background.

We emphasize that use of both MIM and Aria in our curation environment provides expert curators with added flexibility to perform CT segmentation work in either system. This is an important aspect given the multi-campus scope of this project. For example, currently at MCF, ROs tend to perform their segmentation for initial RT planning preparation within MIM, whereas MSR and MCA tend to rely more heavily on Aria tools for segmentation.

#### Automated DICOM De-identification

Careful DeID of DICOM data (i.e., removal of patient identifiers from the DICOM metadata or “tags”) is essential for maintaining patient privacy and this capability was embedded directly into our curation infrastructure. Our DeID requirements were as follows: 1) per institutional guidance, the methods and output needed to strictly follow guidance from the United States Department of Health and Human Services, inclusive of independent Expert Determination (30); 2) we required the ability to customize DeID behavior (in terms of specifying certain operations such as redaction, removal, or replacement to be performed to given DICOM tags, leaving certain tags either totally or partially intact); 3) DICOM objects needed to retain their connectivity (i.e., DICOM object referencing needed to survive DeID translation) and this was achieved by enforcing strict DICOM unique identifier (UID) DeID transformational reproducibility (meaning a given DICOM UID value always mapped to a given DeID UID value) and lastly 4) we needed the DeID process to be traceable (meaning we needed to be able to retrospectively decode which DeID data corresponded to which actual/original patient data).

Given that existing vended solutions for DeID tend to fall short of one or more these requirements, a custom DICOM DeID tool was desired. Fortunately, our IT-Operations team had previously developed a DeID tool meeting these requirements in the context of retrospective studies with our Outcomes Database. For the current project, a new adaptor to this custom DeID tool was built and replicated for each campus-specific curated data storage silo, with each instance allowing patient-identifiable data to flow in from the given MIM-PACS patient list and with scrubbed output data to flow to a given, associated DeID MIM-PACS list (as depicted in **Figure 1**). Critically, the tool can handle multiple DICOM associations (multiple objects and/or multiple patients being transferred from the sending node) and maintains a persistent and query-able database with a published interface (MS Windows application) allowing curators to supply a patient MRN and receive the corresponding DeID-MRN in response.

#### Local network archival

A Mayo Clinic supported NAS was partitioned as our “AISandbox.” Subsequently, our IT Operations team created a high-throughput DICOM adaptor (capable of multiple/parallel DICOM associations) that was connected to MIM to receive outgoing (finalized, curated) DeID data. The output, pushed to the AISandbox from MIM, was organized per DeID patient, with the customized adaptor creating a single parent subdirectory per DeID patient as a container for all DICOM files corresponding to that given patient. This NAS solution is convenient for accessibility across different users and platforms internal to the Mayo Clinic network; related activities may be either within or outside of the scope of curation efforts described herein. We foresee two main use cases, namely 1) long-term curated dataset archival and 2) as a convenient and accessible staging area intermediary to any future automated QA step or methods development.

#### Cloud storage for dataset sharing and collaboration

Due to convenience, accessibility, security and scalability, Cloud storage is the contemporary gold standard for sharing data, whether for applications internal or external to Mayo Clinic. For this project, our external partner requisitioned a storage bucket on a secure CSS for data ingest, which was accessed by Mayo stakeholders using Open Source software.

### Curation workflow

**Figure 2:**
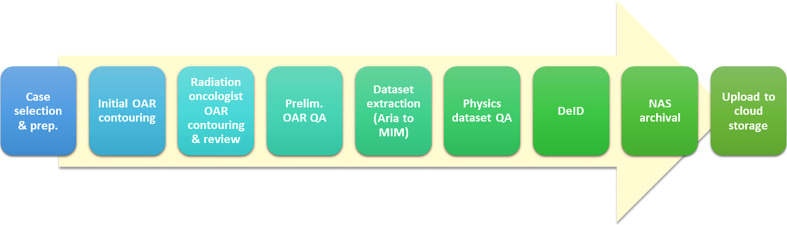
Schematic of dataset curation workflow.

**Figure 2** is illustrative of our current data curation workflow; we will attempt to describe each high-level step in relevant detail. It is important to recognize that this workflow evolved and solidified over the course of the reported experience. Also, there were variations in process related to the provenance of the data – specifically whether it was derived from the MCA or MCR practice. One major difference was the lack of MDA support at MCA. Therefore, for MCA cases prepared for curation in the earlier stages of this H&N project, dosimetrist effort was heavily relied upon; later, th**e** preparation of cases from Arizona was performed by MDAs from the MCR practice.

#### Final case selection; dosimetrist/MDA data preparation and OAR segmentation

In consultation with physicist and physician stakeholders, dosimetrists and/or MDAs examined the output of the raw Outcomes Database query of retrospectively identified candidate H&N cancer patient datasets (as described previously). This selection process, which was carried out in Aria, can be thought of as a careful case-by-case removal of outliers which could bias model training and negatively impact model performance. This involved exclusion of patients that had rare anatomical presentations or anatomy that was significantly altered due to prior clinical intervention. Also, we tended to remove cases with RT planning CT scans that had: 1) missing CT-reconstructions (e.g., missing SFOV reconstruction); 2) non-standard axial CT slice spacing or 3) prominent CT reconstruction artifacts. Whereas, planning CTs with typical CT reconstruction artifacts caused by dental fillings were allowed.

For selected cases, working within the Aria environment, RT planning CT scans were newly organized and appropriately relabeled for better uniformity and consistency. Beginning from copies of the existing, clinically generated structure sets, MDAs or dosimetrists: 1) removed ancillary (also known as “optimization”) structures related to the RT planning process; 2) preserved original clinical (physician-drawn) target structures; 3) reviewed and revised clinical OARs segmentations in light of more recently adopted institution-wide standards, redrawing structures from scratch as needed (if missing or if more efficient than editing existing structures) and 4) made sure labeling across the set of OAR structures was consistent.

As previously discussed, **Table 3** lists the 42 H&N OARs segmented and curated for this project. The dosimetrist from MCR providing data preparation and segmentation effort in the earliest phase of the project included all 42 OARs in their purview. The dosimetrist providing effort for cases derived from MCA included 30 of 42 OARs in their purview (excluding brainstem, brachial plexus, carotid arteries, pharyngeal constrictors, cricopharyngeal inlet, cervical esophagus, nasal cavity, optic nerves and the pituitary gland). MDAs from MCR began to contribute part way into the project. Initially, MDAs had received specialized training to review and segment 38 of the 42 total OARs, excluding only the brachial plexus and carotid arteries from their segmentation scope. During the latter phase, additional MDAs from MCR were recruited to this project and these individuals focused on carotid artery segmentation. For a significant portion of MDA-prepared cases, MDAs kept track of time spent per case in the organization grid. Once preparation work was completed, dosimetrists or MDAs indicated readiness for MD segmentation and review in the organizational grid.

#### Expert radiation oncologist OAR segmentation and review

Once the final curation cohort had been identified and data preparation complete, the H&N expert ROs’ primary effort was focused on reviewing, revising and completing OAR segmentations. As outlined in the previous section, depending on the provenance of the case, some OARs needed to be segmented from scratch. (Oftentimes it was deemed more efficient to start from scratch than to start with copies of OARs derived from the initial clinical RT planning process.) In the organizational grid, MDs added case review notations regarding missing or abnormal anatomy (e.g., due to surgical resection), or any miscellaneous deviations from what might be considered as a standard case presentation (e.g., CT reconstruction artifacts). For a small subset of MDA-prepped cases (N=34), the expert ROs recorded their time spent on this segmentation and review task.

#### Preliminary OAR QA and data extraction

Upon completion of work by expert ROs, MDAs or dosimetrists performed a QA step within Aria prior to data extraction to MIM. For most cases (and all cases toward the end of our H&N curation experience), dosimetrists or MDAs invoked an automated QA step involving an ESAPI script run within the Eclipse environment. Amongst other tests relevant to clinical practice, this script automatically checks for sidedness of bilateral OARs versus their labels (using segmentations for, e.g., brainstem or spinal cord to define the patient’s anatomical midline). It also searches on a per-OAR basis for multiple, discontiguous-2D “parts” which is useful for finding erroneous islands (e.g., tiny “ditzels” in random locations). Relevant findings from this script (which are displayed at run-time in a tabular form) were notated in the organizational grid with erroneous OARs revised if possible. Once this preliminary QA step was completed, data extraction from Aria was invoked for the given case by running the aforementioned Extractavator ESAPI script from within the Eclipse workspace. Dosimetrists or MDAs subsequently indicated completion of data extraction in the organizational grid.

#### Physics curated dataset QA

Upon completion of OAR segmentation and extraction from Aria, expert physicists performed a final, comprehensive QA review applied to the entirety of the curated dataset. This step was carried out within the MIM environment and was both facilitated and safeguarded by having partitioned MIM-PACS “patient lists” dedicated to this curation effort. To summarize, the three main foci of this physics QA step were 1) verification of presence of intended/required DICOM objects; 2) verification of capture of clinical RT plan objects and 3) final QA of expert-curated OAR segmentations. Each of these is described in more detail below:

##### DICOM object QA

The goal of this step was to ensure completeness of the curated dataset, per the definition in **Table 1**. Certain logic had been coded into the Extractavator to search Aria for SFOV and contrast CT reconstructions; the SFOV CT always shares a DICOM “Frame-of-Reference” with the planning CT, whereas the contrast CT is sometimes alternatively linked to the planning CT *via* a DICOM Spatial Registration object (i.e., CT image “fusion,” dependent on workflow during CT simulation). Issues with data completeness sometimes arose for unexpected labeling nomenclature in Aria, or in cases where certain CT reconstructions had not been originally included in Aria for clinical RT management. For some cases, it was necessary to perform additional manual DICOM extractions from Aria or from our institutional DICOM PACs archive.

##### DICOM RT-Plan QA

Since this aspect is out of scope for this report, we will describe this step at a high level only. Our intent was to capture the treated RT plan or plans pertaining to each curated case. This was a nuanced objective given the propensity for multiple clinical RT plans being generated (either in the case of a sequential boost or in the case of a plan adaptation being performed part way through treatment). The Extractavator referred to DICOM Frame-of-Reference as well as Aria plan status (pertaining to “treatment approval” and also requiring a record of RT delivery) in order to determine which RT plans were relevant for extraction. Given this nuance, our pragmatic working goal was to package the necessary set of DICOM-RT Plans with their RT-Dose objects that would best reflect the intended course of RT, as opposed to reflecting “the actual” course of RT (which may have involved other DICOM planning CTs and hence other DICOM coordinate systems).

##### Curated RT-Struct QA

A “Contour QA” MIM Workflow was supplied by engineers at MIM Software, Inc. to be evaluated in the context of either routine physics plan check or physician peer review. Originally, this Workflow contained support for both candidate-problem detection as well as for interactive correction. We modified the Workflow to remove the latter interactive repair aspect and standardized the location where the candidate-problem report (.csv-formatted table) would be saved.

This Workflow performs five checks per given labeled structure, namely: 1) whether the structure is empty (contains no actual 2D contours); 2) whether any part of the structure is outside the defined “body” or “external” structure (which is a required segmentation that is used by RT planning systems to define the bounds of the dose calculation or relevant patient extent); 3) whether the structure contains “holes” (areas of exclusion, such as in a doughnut shape); 4) whether the structure contains multiple (discontiguous) parts; and 5) whether detected sidedness is consistent with the structure label. Note, these latter two are same or similar checks as were performed in Aria with our previously described ESAPI QA tool. Checks 3 and 4 were subject to a user-defined volume threshold which was hardcoded at 0.003 cc. This volume threshold was smaller than that used in the previously described ESAPI tool, making it more sensitive to finding tiny holes or islands in the structure (“ditzels”). The Workflow recorded each per-structure test result as a “PASS,” “FAIL” or “N/A” in the output .csv report. These reports were copied to curator-accessible cloud storage with corresponding file hyperlinks embedded inside the organizational grid (in the row corresponding to the given patient and in a column dedicated for this MIM Contour QA step). We note that with our distributed Citrix environment this Workflow required approximately 5 minutes to run per case; given our per-user random-access memory allocation (approximately 20 GB) we were able to run the Workflow on two patients’ DICOM-RT Structs simultaneously (in separate MIM “sessions”).

Subsequently, the generated Contour QA report was reviewed manually by the expert physicist with the DICOM planning CT and DICOM RT-Struct being reviewed simultaneously within the MIM application. Depending on the test, false failures were observed to occur with relatively high frequency. For example, the segmentations corresponding to the mastoid and brachial plexus commonly failed the multiple-parts test. A hole failure was common for lung segmentations due to airways and vessels. True failures won’t necessarily contribute to problems with AI modeling downstream. "Ditzels" are difficult to find manually in context and often result from accidental “mouse clicks” or from conversion to DICOM from Aria’s native structure set format. Since these tend to be unrelated to the underlying anatomy or image and are stochastic (i.e., non-reproducible), they likely have insignificant impact on AI model training. True failures which we interpreted as needing correction, as well as any other problems incidentally observed during manual interactive OAR segmentation review in MIM, were noted directly in the .csv report with a succinct description of the issue. An indication of whether issues were found in this process was provided by changing the color of the .csv report link in the organizational grid to either green (for pass) or red (for fail).

This organization allowed other expert curator stakeholders to re-engage with OAR corrections. In our workflow, initially dosimetrists, and later MDAs, performed the step of resolving any potential OAR deficiencies or errors. In some cases, an expert radiation oncologists’ re-review was required. We opted to maintain the same “final” curated structure set in both the MIM and Aria environments.

#### De-identification

Upon the completion of the above, data underwent DeID. In our infrastructure, this required the curator to select the given patient(s’) record(s) in MIM (all associated DICOM objects contained in the given MIM-PACS patient list) and export them to a defined DICOM node (daemon) corresponding to the custom DeID tool described previously. Supporting parallelization *via* multiple DICOM associations, this tool could handle DICOM objects from multiple patients being sent simultaneously. Subsequently, the DeID tool’s output was automatically forwarded to a separate MIM-PACS patient list designated for finalized, DeID data. To verify completeness of this step, we queried the DeID tool’s database using the previously mentioned custom Windows application by supplying the original patients’ MRN and receiving back the DeID patients’ MRN (DeID-MRN). We note that multiple MRNs could be handled in this fashion *via* a single query. Accordingly, the DeID-MRNs were recorded in our data organization grid. Thus, the MIM-PACS patient list corresponding to the DeID patients could be searched and reviewed for curated object correctness and completeness.

#### NAS archival

Following DeID, finalized curated datasets were extracted from our MIM-PACS environment to our dedicated AISandbox NAS using the custom DICOM adaptor described previously. For convenience, this step tended to be completed in multi-patient mini-batches using MIM’s built-in “advanced” DICOM query capability which allows multiple DeID-MRNs to be supplied *via* regular-expression (“regex”) syntax. For this project, stored on the NAS along with the curated dataset archive was relevant redacted and DeID demographic information along with ascii-formatted “manifests” of DeID patients as well as a full DICOM-object list (as represented by DICOM SOPInstanceUIDs).

#### Upload to cloud storage

In the final step, finalized, DeID curated datasets were uploaded in bulk at high bandwidth to the secure CSS from the AISandbox NAS using an Open-Source, multi-threaded data transfer utility.

## Results

**Figure 3:**
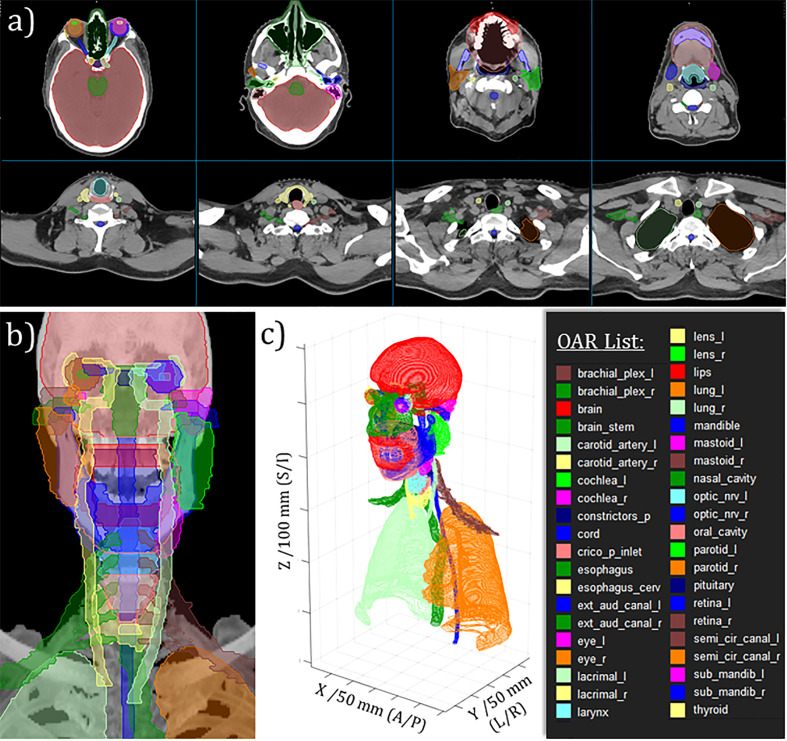
Example of curated H&N OAR segmentations for randomly selected case. **(A)** Moving from left-to-right, top-to-bottom: selected axial slices moving from superior to inferior aspect of H&N RT planning CT. **(B)** Anterio-posterior maximum intensity projection rendering with OAR projections overlayed. **(C)** 3D perspective rendering of 2D DICOM-RTStruct-format OARs with tick-marked scales on DICOM coordinates as indicated.

Over approximately one year, from August 2020 through September 2021, our expert multidisciplinary team generated 490 curated H&N datasets. A randomly selected case is shown in **Figure 3**, which demonstrates the complexity of the H&N anatomy and delicate nature of the OAR segmentation task. As shown by individual rows in **Table 4**, the datasets were subdivided into four separate cohorts, wherein there is no relevant delineation between cohorts 1-3. The cohort labeled as “Hold Out” (HO) was withheld from our external partner to enable us to retain explicit independence in terms of retrospective evaluation of DL model performance. This provided a separate means for DL model validation, over and above standard validation and testing processes associated with the DL model training itself. We also mention that the HO cohort was slightly different from the other curated data in that we intentionally enriched its diversity giving more careful inclusion consideration for ethnicity or race and sex. The HO cohort was also well balanced for primary tumor site and primary RT versus postoperative RT.

**Table 4:** Mean, median and standard deviation of time spent per recorded case by H&N anatomy experts for OAR segmentation and related revisions, broken down per cohort subset.

			MDA carotid time (min.)				MDA other OAR time (hr.)				Physician time (hr.)		
Cohort	N_Cases_	N_Samples_	mean	median	σ	N_Samples_	mean	median	σ	N_Samples_	mean	median	σ
1	251	28	27.7	27.5	6.6	63	6.9	6.00	2.9	-	-	-	-
2	105	39	24.1	20.0	9.2	72	7.1	7.00	0.6	-	-	-	-
3	99	74	35.3	35.0	8.7	77	7.4	7.50	0.6	-	-	-	-
Hold Out	35	-	-	-	-	35	6.6	6.75	0.8	34	4.0	4.25	1.6
Combined	490	141	30.7	30.0	9.8	247	7.1	7.00	1.6	34	4.0	4.25	1.6

MDA time spent on carotid segmentation was recorded to the nearest 5 minutes; time spent by MDAs on other OARs, as well as physician time, was recorded to the nearest 15 minutes. MDA effort can be considered as interchangeable with dosimetrist effort.

**Figure 4:**
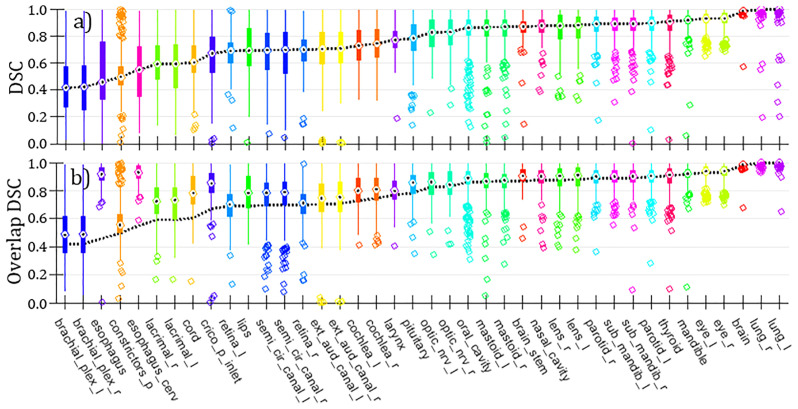
**(A)** Box and whisker plots of DSC comparing curated versus clinical H&N OARs (N_Cases_=490; counts per OAR are variable, dependent on set of OARs available per case). “Bulls-eyes” indicate the median values; medians are connected over all OARs with a dashed line. **(B)** Same representation as in panel **(A)** for alternative metric Overlap DSC. The dashed line showing medians from (standard) DSC (taken from panel) **(A)** is projected onto this panel for comparison.

**Figure 4A** is a summary of volumetric Dice Similarity Coefficient (DSC, also known as Sørensen–Dice Coefficient) comparing curated versus clinical OARs over the entire set of H&N curated cases (N_Cases_=490). We note that the statistics per OARs in **Figure 4** are variable to due to absence of given OARs in either structure set (primarily the clinical structure set). DSC is a standard quantitative volume metric describing OAR geometric overlap and is defined on the range [0-1] with 1 corresponding to 100% of voxels overlapping and 0 corresponding to no voxels overlapping (23, 31). (DSC tends to overemphasize differences in small or complex OARs while being relatively insensitive to differences for larger OARs.) Median DSC ranged from approximately [0.4-1] over the set of curated OARs, with significant variance in per-OAR DSC distributions observed. Carotid arteries are absent because they are missing from the clinical OARs. This finding, notably poor DSC correspondence for a significant proportion of OARs, helps to underscore the importance of using consistent and standardized (i.e., carefully curated) OAR segmentations rather than retrospective clinical OAR segmentations for DL model training. Contributing to the observed mismatches are certainly 1) inter-observer variation; 2) changes in H&N contouring guidelines over time; and (related to this) 3) systematic practice differences between MCR and MCA. **Figure 4B** is a comparison using a similar metric we have dubbed “Overlap DSC” which limits the respective volume calculation to CT slices that have contours for *both* OARs under comparison. Given this definition, Overlap DSC minimizes bias to changing OAR definitions in terms of segmented superior-inferior extent. Four OARs, namely esophagus, esophagus_cerv (cervical esophagus), cord (spinal cord) and crico_p_inlet (cricopharyngeal inlet muscle) stand out as likely having poor (standard) DSC due to an evolution in our institutional definition for OAR extent.

### Time spent per curated case

It is extremely relevant to report our experience in terms of human effort required per curated H&N dataset. Accurate time recording was available for a subset of cases; this is summarized in **Table 4**. From this, using median values and their standard deviations (added in quadrature), we can estimate that initial OAR segmentation and review by MDAs (or dosimetrists) and subsequently physicians required approximately 11.3 ( ± 2.3) hours per case (assuming MDAs did not assist with carotid segmentation, as in the HO cohort).

Based on anecdotal experience, expert physics processing, QA review and DeID required 30 minutes per case at a minimum, also assuming no downstream OAR corrections were needed. It is reasonable to augment this towards an overall QA and dataset organization time requirement of approximately 1 hour per case considering downstream corrections, dataset navigation through the curation pipeline and subsequent organizational efforts such as cohort aggregation and archival. In addition, there was substantial time and effort devoted to planning and development of the underlying infrastructure as well as for training personnel; these inputs are generally difficult to accurately account for.

### Frequency of OAR candidate-problems identified by physics QA

Expert physics review and QA of curated H&N datasets was likely an important step in the curation process. Retrospective review of our data organization grid revealed that an expert physicist flagged OAR segmentation issues in 170 out of 439 cases (39%) for further review and potential revision. The majority of these likely would have had minor, if not insignificant, impact on model training (dependent to an extent on our external partner’s OAR QA procedures). Amongst the more significant, but infrequent, errors detected were left vs. right sidedness mislabeling, as well as accidental inclusion of segments intended for one OAR as part of another OAR. The automated MIM Contour QA Workflow made a substantive impact in helping to flag these more significant issues. We note that the above tally of physics QA interventions excludes helpful inputs from other semi-automated QA methods currently under development that involve parsing DICOM data post-extraction to our AISandbox NAS archive.

## Discussion

Our study demonstrates the extent of thought, planning, novel infrastructure development and human effort that is required to conduct an RT data curation project at this level of quality and at large scale. This has significant practical value for other investigators or institutions planning to embark on similar projects. It is difficult to find reports like ours in the literature. Our review uncovered one prior study pertaining to curation infrastructure for image labeling in the context of radiology (32) and another prior study related to retrospective curation of breast DICOM-RT data for downstream correlation with dosimetric endpoints (33). Somewhat related, Wong et al. described their RT patient selection and expert OAR segmentation efforts towards DL-based autosegmentation model evaluation, inclusive of capturing inter-expert variability (20). In a later report, the same institution described their expert OAR dataset preparation for model training and validation for a different anatomical site (21). The general sparsity of prior reporting may be indicative of a difficult peer review barrier (or perhaps a perception of a difficult peer review barrier) – the potential challenges being that this and similar reports could be easily discarded as either non-hypothesis-driven or non-hypothesis-generating, or that they might be discounted under micro-dissection due to lack of novelty of constituent methods. However, when viewed under a practical or macroscopic lens, we assert that this report is substantial, unique and adds value to our RT knowledge base.

Our specific findings in terms of per-case time requirements for generation of 42 expert H&N OAR segmentations of >11 hours per case (on average) are important. In the context of a study on clinical validation of atlas-based autosegmentation model performance, Teguh et al. mention that generation of 20 expert H&N OARs (submandibular glands, parotid glands, chewing and swallowing muscles, spinal cord) in ten patients required 3 hours per case (on average) (34). Wong et al. report that preparation of expert H&N OARs (spinal cord, parotid glands, submandibular glands, inclusive of neck CTV) required 26.6 minutes on average (20). In a review of studies reporting time savings from H&N autosegmentation for H&N RT planning, Lim and Leech summarize initial H&N OAR segmentation time as requiring 2.7-3 hours (35). The relevance of comparing these prior studies to our findings is questionable given the much larger set of OARs generated in our H&N curation example and the specific contexts around time reporting elsewhere. Nevertheless, we emphasize here that expert H&N OAR segmentation is clearly a time-consuming process.

In our view, the curated datasets generated in this study have potentially high value for other applications besides DL-based model training. For example, the expert OARs can be used for: 1) benchmarking existing autosegmentation models/solutions against our defined clinical standards; 2) benchmarking clinician/RO OAR segmentation performance across clinical practice; or 3) creating independent H&N OAR QA tools based on feature extraction and machine learning – not forcibly for autosegmentation but rather for verifying OAR consistency on future clinical H&N planning CTs or used as part of an automated watchdog reviewing output of autosegmentation models (8–10, 36). With the H&N datasets generated in this study, we have already done extensive work on example 1 and begun exploratory work on example 3.

It must be emphasized that the infrastructure created specifically under the scope of our first H&N dataset curation use case has generalizable utility which can easily scale, whether for larger or smaller RT dataset curation exercises. Work is currently ongoing to curate datasets for brain RT with new stakeholders (physicists and ROs) engaged in this latest effort from across all Mayo campuses; for these efforts a portion of the curated datasets will also be derived from MCF. Planning for other body sites is also underway. More broadly speaking, this infrastructure could support numerous DICOM-RT data driven projects inclusive of clinical trials QA or research. The key scalable and recyclable elements of this infrastructure are: 1) MIM, as central to conveniently accessing, visualizing or manipulating the DICOM-RT data and supporting layers of automation; 2) automated or semi-automated data extraction from the RT information system (Aria) to MIM (i.e., the Extractavator); 3) automated DICOM daemons for DeID and DICOM-RT dataset extraction from MIM; and 4) archival DICOM-RT storage (either NAS or cloud-based, or both). A potential wish or need is for an ancillary DICOM storage web service (“DICOM store”) providing a convenient ingest or egress capability for DICOM-RT data, facilitating seamless collaboration with external stakeholders. As an example, MIM Software deploys MIMCloud™ as a high-value Cloud-based companion DICOM store for MIM Maestro.

## Conclusion

Mayo Clinic has recently embarked on a large-scale and interdisciplinary RT dataset curation effort pertaining to DL-based autosegmentation of OARs. In support of this endeavor, we constructed a scalable, semi-automated DICOM-RT dataset pipeline for data extraction, visualization, QA, DeID, long-term archival and dataset sharing. We demonstrated functionality of this infrastructure, reporting here on our first experience leveraging these tools for curation of 490 H&N RT datasets. This required significant human-expert effort, estimated at greater than 12 hours per case. Due to the well-designed infrastructure (which automated otherwise extremely tedious and time-consuming steps), the bulk of this effort was able to be focused on OAR segmentation, related review and QA, which are aspects truly requiring human expertise.

## Data availability statement

The datasets presented in this article are not readily available because of Mayo Clinic policies on data use and sharing. Requests to access the datasets should be directed to tryggestad.erik@mayo.edu.

## Author contributions

All authors contributed to this article in some fashion. Author ET (corresponding author) was primarily responsible for conception, design, content and writing of this manuscript. The dataset curation infrastructure we report on was primarily designed and constructed by authors ET, SSe and DJM. The H&N dataset curation efforts summarized in this manuscript was split across all authors, with AH, SHP, RLF, ET, AA, DJM, NNL and CB providing support and supervision of the process. Senior author RLF was the H&N radiation oncologist expert who segmented and/or reviewed the largest proportion of the curated H&N datasets. All authors approved the submitted article.

## Acknowledgments

We wish to thank Mayo Clinic Information Technology, specifically the Radiation Oncology Operations and Development teams who supported this project and tirelessly support our RT computing and networking infrastructure at large. We acknowledge our colleague Dr. David Ellerbusch who authored the contour QA ESAPI tool used by dosimetrists and MDAs. We also thank the skillful and collaborative engineering team at MIM Software who initially provided a version of the contour QA MIM Workflow leveraged here. Lastly, we acknowledge the supportive environment at Mayo Clinic in the Department of Radiation Oncology and, in particular, wish to thank our executive leadership team.

## Conflict of interest

The authors declare that the research was conducted in the absence of any commercial or financial relationships that could be construed as a potential conflict of interest.

## Publisher’s note

All claims expressed in this article are solely those of the authors and do not necessarily represent those of their affiliated organizations, or those of the publisher, the editors and the reviewers. Any product that may be evaluated in this article, or claim that may be made by its manufacturer, is not guaranteed or endorsed by the publisher.

## Abbreviation

MCR, Mayo Clinic Rochester; MCF, Mayo Clinic Arizona; MCF, Mayo Clinic Florida; CSS, cloud storage service; RT, radiotherapy, or radiation therapy; AI, artificial intelligence; ML, machine learning; QA, quality assurance; OAR, organ-at-risk; H&N, head & neck; RO, radiation oncologist; MDA, medical dosimetry assistant; SFOV, small field-of-view; DeID, de-identified, or de-identification; NAS, Network-Attached storage; PACS, Picture Archiving and Communications System; MS, Microsoft™; API, application programming interface; UID, unique identifier; MRN, medical record number.
